# Supplementary material for: Helminth infection induces RELMα-dependent adipose tissue transcriptional reprogramming and protection against diet-induced obesity
Source: bioRxiv. 2026 Jun 9:2026.06.04.730169. Preprint. [Version 1] doi: 10.64898/2026.06.04.730169 (PMC13278041; doi:10.64898/2026.06.04.730169)
Supplement: Supplementary file 1 [file NIHPP2026.06.04.730169v1-supplement-1.pdf]

bioRxiv preprint doi: https://doi.org/10.64898/2026.06.04.730169; this version posted June 9, 2026. The copyright holder for this preprint (which was not certified by peer review) is the author/funder, who has granted bioRxiv a license to display the preprint in perpetuity. It is made available under a CC-BY 4.0 International license.

Supplemental material

***Supplemental Figure 1*. Representative gating strategy for analysis of bronchoalveolar lavage fluid immune cells.**

(A) Representative flow cytometry plots showing the gating strategy used to identify alveolar macrophages and eosinophils in bronchoalveolar lavage fluid (BAL). Events were sequentially gated on cells, singlets, and CD45□ immune cells. Alveolar macrophages were defined as CD11c□Siglec-F□ cells and eosinophils as CD11c□Siglec-F□ cells within the CD45□ population.

***Supplemental Figure 2.* Western diet increases parasite egg burden following infection.**

(A) Quantification of parasite egg burden from WT and RELMα KO male mice maintained on CD or WD for 10 weeks prior to infection with 550 *Nippostrongylus brasiliensis* larvae per mouse. Egg burden was assessed at day 7 post-infection. Data are presented as group means ± SEM. Statistical analysis was performed as described in Methods. Representative of 2 independent experiments, n =3–5 mice per group per experiment.

***Supplemental Figure 3.* Differential gene expression analysis of adipose tissue from naïve WT versus naïve KO mice and naïve KO versus infected KO mice.**

(A) Differential gene expression analysis of naïve WT versus naïve KO adipose tissue performed using DESeq2. Genes meeting criteria of absolute log2 fold change ≥ 0.25 and adjusted $p \leq 0.05$ are shown. **(B)** Differential gene expression analysis of naïve KO versus infected KO adipose tissue performed using DESeq2. Genes meeting criteria of absolute log2 fold change ≥ 0.25 and

bioRxiv preprint doi: https://doi.org/10.64898/2026.06.04.730169; this version posted June 9, 2026. The copyright holder for this preprint (which was not certified by peer review) is the author/funder, who has granted bioRxiv a license to display the preprint in perpetuity. It is made available under a CC-BY 4.0 International license.

adjusted $p \leq 0.05$ are shown. Representative of 1 experiment, $n = 3$ to 4 mice per group.

bioRxiv preprint doi: https://doi.org/10.64898/2026.06.04.730169; this version posted June 9, 2026. The copyright holder for this preprint (which was not certified by peer review) is the author/funder, who has granted bioRxiv a license to display the preprint in perpetuity. It is made available under a CC-BY 4.0 International license.

Supplemental Table 1. BALF Flow Cytometry Panel

Laser	Fluorophore	Surface	Company	Cat.	Clone
**488 nm**	FITC	F4/80	Biolegend	123108	BM8
	PerCP, 7AAD	7AAD	Biolegend	420404	N/A
	PerCP Cy5.5	CD115	Biolegend	135526	AFS98
	PE Cy7	Ly6C	Invitrogen	25-5932-82	HK1.4
**637 nm**	APC	CX3CR1	Biolegend	149008	SA011F11
	AF700	MHCII	Invitrogen	50-169-17	M5/114.15.2
	APC Cy7	CD11b	Biolegend	101226	M1/70
**405 nm**	Pacific Blue, BV421	CD163	Biolegend	155309	S15049I
	AmCyan, BV421	Ly6G	Biolegend	127627	1A8
	Pacific Orange, BV570	CD45	Biolegend	103135	30-F11
	Qdot 605, BV605	CD64	Biolegend	139323	X54-5/7.1
	BV711	CD206	Biolegend	141727	C068C2
**561 nm**	PE, Td Tomato	CCR2	Biolegend	150610	SA203G11
	PE Texas Red, PE Dazzle	SiglecF	Biolegend	155530	S17007L
	PE Cy5.5	CD11c	Invitrogen	35-0116-42	3.9

bioRxiv preprint doi: https://doi.org/10.64898/2026.06.04.730169; this version posted June 9, 2026. The copyright holder for this preprint (which was not certified by peer review) is the author/funder, who has granted bioRxiv a license to display the preprint in perpetuity. It is made available under a CC-BY 4.0 International license.

Supplemental Table 2. Adipose Immunofluorescence Panel

Primary	Company	Cat.	Clone
GSL I Isolectin B4, Biotinylated	Vector Laboratories	B-1205-.5	N/A
Rat anti-mouse RELMa	Invitrogen	MA5-23898	226033
Rat anti-mouse Lyve1, eBioscience	Invitrogen	14-0443-82	ALY7
Rabbit anti-mouse Serpine1	Abcam	AB28207	N/A
Rabbit anti-mouse COL6A1	Proteintech	17023-1-AP	N/A
**Secondary**	**Company**	**Cat.**	**Clone**
SAV AF488	Invitrogen	S32354	N/A
Goat anti-Rat Cy3	Invitrogen	A10522	N/A
Goat anti-Rabbit Cy5	KPL	072-02-15-06	N/A
